# Endothelial Cell Phenotype, a Major Determinant of Venous Thrombo-Inflammation

**DOI:** 10.3389/fcvm.2022.864735

**Published:** 2022-04-21

**Authors:** Marion Pilard, Estelle L. Ollivier, Virginie Gourdou-Latyszenok, Francis Couturaud, Catherine A. Lemarié

**Affiliations:** Inserm, Univ Brest, CHRU Brest, UMR 1304, GETBO, Brest, France

**Keywords:** venous thromboembolism, endothelial cell, inflammation, endothelial plasticity, fibrosis

## Abstract

Reduced blood flow velocity in the vein triggers inflammation and is associated with the release into the extracellular space of alarmins or damage-associated molecular patterns (DAMPs). These molecules include extracellular nucleic acids, extracellular purinergic nucleotides (ATP, ADP), cytokines and extracellular HMGB1. They are recognized as a danger signal by immune cells, platelets and endothelial cells. Hence, endothelial cells are capable of sensing environmental cues through a wide variety of receptors expressed at the plasma membrane. The endothelium is then responding by expressing pro-coagulant proteins, including tissue factor, and inflammatory molecules such as cytokines and chemokines involved in the recruitment and activation of platelets and leukocytes. This ultimately leads to thrombosis, which is an active pro-inflammatory process, tightly regulated, that needs to be properly resolved to avoid further vascular damages. These mechanisms are often dysregulated, which promote fibrinolysis defects, activation of the immune system and irreversible vascular damages further contributing to thrombotic and inflammatory processes. The concept of thrombo-inflammation is now widely used to describe the complex interactions between the coagulation and inflammation in various cardiovascular diseases. In endothelial cells, activating signals converge to multiple intracellular pathways leading to phenotypical changes turning them into inflammatory-like cells. Accumulating evidence suggest that endothelial to mesenchymal transition (EndMT) may be a major mechanism of endothelial dysfunction induced during inflammation and thrombosis. EndMT is a biological process where endothelial cells lose their endothelial characteristics and acquire mesenchymal markers and functions. Endothelial dysfunction might play a central role in orchestrating and amplifying thrombo-inflammation thought induction of EndMT processes. Mechanisms regulating endothelial dysfunction have been only partially uncovered in the context of thrombotic diseases. In the present review, we focus on the importance of the endothelial phenotype and discuss how endothelial plasticity may regulate the interplay between thrombosis and inflammation. We discuss how the endothelial cells are sensing and responding to environmental cues and contribute to thrombo-inflammation with a particular focus on venous thromboembolism (VTE). A better understanding of the precise mechanisms involved and the specific role of endothelial cells is needed to characterize VTE incidence and address the risk of recurrent VTE and its sequelae.

## Introduction

A growing body of evidence reveals the functional interdependence of inflammation and thrombosis in cardiovascular diseases. The intricate relationship between these two processes, where inflammation begets thrombosis, and in turn, thrombosis amplifies inflammation, is mediated by the endothelium, leukocytes and platelets in a process termed immuno-thrombosis (1). In physiological conditions, activation of the coagulation cascade by inflammation is part of a natural defense mechanism against pathogens. However, it now well established that similar mechanisms are involved in aberrant activation of inflammatory-dependent thrombosis. This process can occur in sterile conditions and is characterized by a cascade of signals leading to the recruitment and activation of neutrophils, monocytes and platelets that is partially orchestrated by the endothelium (2). The integrity of the endothelium is an important protective mechanism that ensures circulatory homeostasis and prevents thrombus formation. Endothelial dysfunction is characterized by an imbalance between pro- and anti-coagulant factors and between pro- and anti-inflammatory mediators (3). This is directly responsible for various cardiovascular diseases including hypertension, atherosclerosis, stroke, heart disease, diabetes, pulmonary arterial hypertension and venous thrombosis. Recent studies have better characterized one specific mechanism, namely the endothelial-to-mesenchymal transition (EndMT), contributing to endothelial dysfunction during inflammation (4). EndMT is a complex mechanism leading to phenotypic switching of the endothelium associated with acquisition of mesenchymal makers and properties leading to pathological states including tissues fibrosis. EndMT occurs consequently to activation of endothelial cells by pro-inflammatory factors including cytokines but also to changes in the environment such as hypoxia and released in the extracellular space of damage-associated molecular patterns (DAMPs). Endothelial cells are equipped with specific receptors and sensors allowing them to respond to these signals and consequently adapt their phenotype. Here, we discuss how the endothelial phenotype is central to the interactions between inflammation and thrombosis in cardiovascular diseases with a general focus on venous thromboembolism (VTE). Better characterization of these processes will be crucial to prevent recurrent events and long-term complications associated with VTE.

## VTE and Sterile Inflammation

VTE, which encompasses deep venous thrombosis (DVT) and pulmonary embolism (PE), is a frequent and life-threatening disease. VTE is the third leading cause of cardiovascular death after myocardial infarction and stroke with a mortality rate of 10% at 3 months after PE. VTE affects 1 to 2 per 1,000 persons per year and is associated with long-term complications affecting the life expectancy and the quality of life of patients. Main complications of VTE include recurrent (non-fatal and fatal) VTE and long-term sequelae due to incomplete clot resolution in the leg veins [the post-thrombotic syndrome (PTS)] and/or in pulmonary arteries [chronic thromboembolic disease [CTED] and chronic thromboembolic pulmonary hypertension (CTEPH)]. Hence, VTE is a major health issue representing 240 million euros per year in France (5, 6).

**Figure 1:**
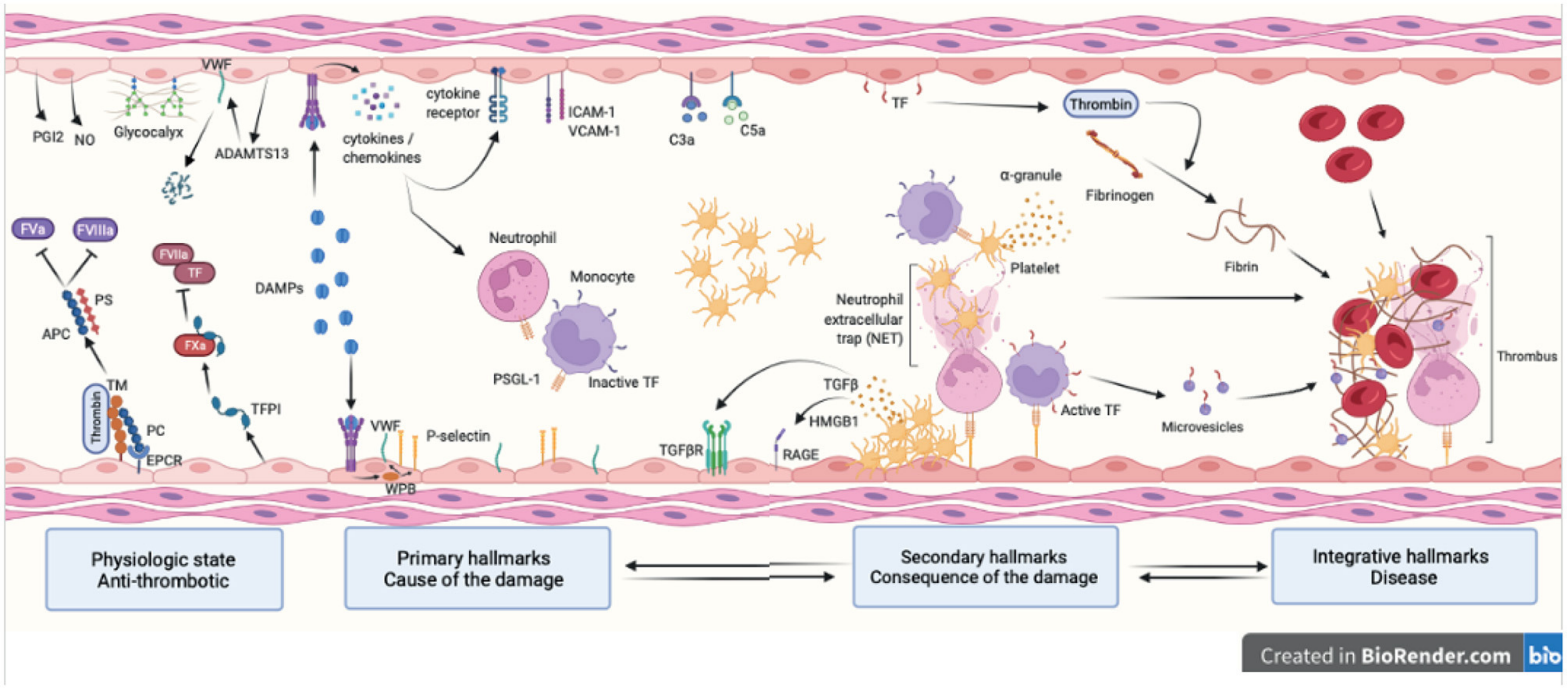
Hallmarks of venous thromboembolism. Healthy endothelial cells provide an anti-coagulant surface by expressing anti-coagulant factors (TM, TFPI, EPCR and PC) limiting thrombin generation. Intact glycocalyx and production of NO and PGI_2_ also protect against venous thrombosis. Thrombo-inflammation is triggered by the release in the bloodstream of DAMPs following cell injury. DAMPs interact with the endothelium and promote the release of cytokines, chemokines and WPB content and expression of adhesion molecules (primary hallmarks). This leads to endothelial dysfunction characterized by platelet and leukocyte recruitment that will in turns become activated and secrete pro-inflammatory and pro-coagulant molecules further contributing to thrombosis (secondary hallmarks). Together with the complement system, platelets and endothelial cells induce NET formation and TF expression in monocytes through interactions between P-selectin and PSGL-1. This initiates the coagulation cascade through both the intrinsic and extrinsic pathways and ultimately leads to thrombin-induced fibrin generation and formation of an obstructive clot (integrative hallmarks). APC, activated protein C; TM, thrombomodulin; TFPI, tissue factor pathway inhibitor; EPCR, endothelial protein C receptor; PC, protein C; NO, nitric oxide; PGI_2_, prostaglandin I 2; DAMP, damage-associated molecular pattern; WPB, Weibel Palade body; NET, neutrophil extracellular trap; TF, tissue factor; PSGL-1, P-selectin glycoprotein ligand-1; RAGE, receptor for advanced glycation endproducts; HMGB-1, high mobility group box-1.

VTE is a multifactorial and complex disease involving individual predispositions, environmental parameters and genetic determinants. It is defined as an exaggerated hemostatic response, leading to the formation of an occlusive blood clot obliterating blood flow in the venous system (7). Virchow's triad was traditionally invoked to describe pathophysiological mechanisms fostering thrombosis, alleging concerted roles for hypercoagulability, endothelial dysfunction, and hemodynamic changes (stasis and turbulence) in the development of venous thrombosis (8). It is now well established that sterile inflammation drives VTE formation and resolution (Figure 1).

Sterile inflammation occurs in acute conditions including ischemia reperfusion injury and trauma and depends on a well-orchestrated migration sequence of leukocytes to and from the site of injury (9). Sterile inflammation refers to inflammation triggered by tissue injury in the absence of infection. It causes reactive oxygen species (ROS), cytokines, chemokines and DAMP production following endothelial, platelet and leukocyte interactions. Consequently, necrotic events are initiated leading to the release of self-DAMPs including nucleic acids, high-mobility group box 1 (HMGB1), heat shock proteins and purine metabolites such as ATP (10). The recruitment of neutrophils and monocytes at early stages and probably lymphocytes at latter stages is in part orchestrated by the endothelium through cytokine secretion and adhesion molecule expression. Endothelial dysfunction plays a crucial role in VTE, both in thrombus formation and resolution. The endothelium is a major regulator of vascular homeostasis. Its main function is to form a barrier controlling the transport of molecules and cells between the bloodstream and the vessel wall. In addition, under physiological conditions, the endothelium responds to a series of chemical and biomechanical cues by secreting factors regulating vascular tone, smooth muscle cells proliferation and migration, thus preventing immune cell adhesion, thrombosis and inflammation. Endothelial dysfunction is an early predictor of subsequent cardiovascular events or mortality including in VTE (11). Hence, it has been associated with spontaneous VTE in patients further highlighting the importance of the endothelial phenotype in modulating thrombo-inflammatory events (12).

## Endothelial Dysfunction and Mechanisms of Thrombo-Inflammation

### Homeostasis

Endothelial cells form a monolayer called the endothelium covering the inner surface of blood vessels. When discovered in 1865, the endothelium was described as an inert barrier between blood and tissues. However, it is now well recognized that endothelial cells are capable of plasticity and phenotypical changes according to the microenvironment. At the whole-organism level, endothelial cells share essential functions in a multitude of physiological processes. They regulate vasomotor tone, vascular permeability, angiogenesis, immune cell trafficking, inflammation, coagulation and transport of nutrients, hormones, growth factors and particles. Importantly, the endothelium also exerts a number of organ-specific functions which conditioned the heterogeneity of endothelial cells (13). However, when dysfunctional, they strongly contribute to the pathogenesis of cardiovascular disease, including VTE (14).

In physiological conditions, endothelial cells guarantee an anti-coagulant and anti-inflammatory state through the integration of complex mechanisms to avoid contact between proteins from the coagulation cascade and blood cells. An intact endothelium actively regulates the coagulation response through potent inhibitory processes. Endothelial cells express various anticoagulant molecules, such as tissue factor pathway inhibitor (TFPI), thrombomodulin (TM), endothelial protein C receptor (EPCR), and heparin-like proteoglycans constituting the glycocalyx. The endothelium also expresses ectonucleoside triphosphate diphosphohydrolase-1 (ENTPDase1/CD39) which metabolizes ATP into adenosine preventing platelet activation and aggregation (15). Other mechanisms include endothelial synthesis of nitric oxide (NO) and prostacyclin I2 (PGI_2_). These molecules inhibit platelet adhesion and aggregation and vasodilation of the vessel (16).

TFPI limits the action of tissue factor (TF), responsible for the activation of the extrinsic pathway. TFPI acts via factor Xa (FXa) to inhibit the formation of the TF-FVIIa complex (17). In addition, the TM/thrombin complex binds and activates protein C, which in turns binds to protein S and inactivates FVa and FVIIIa (18). Endothelial cells also regulate coagulation by producing the serine proteases, tPA (tissue-type plasminogen activator) and uPA (urokinase-type plasminogen activator) that cleave plasminogen into plasmin (19, 20). Finally, the glycocalyx, which is negatively charged, repels negative molecules thus preventing activation of the coagulation cascade (21). The glycocalyx also fosters the formation of complexes between antithrombin III and thrombin (factor II) or with other serine proteases (factor IXa, Xa, XIa and XIIa) (22).

Additionally, the glycocalyx provides the endothelium with a negatively charged surface contributing to the anti-adhesive properties of the endothelial cell surface. Initial platelet adhesion to VWF is also prevented by a disintegrin-like and metalloprotease with thrombospondin type I repeats−13 (ADAMTS13) cleaving VWF multimers released from the Weibel-Palade bodies (WPBs) (23, 24). *In vivo* studies using experimental models of inflammation showed that ADAMTS13 deficiency is associated with enhanced WPB secretion and increased leukocyte adhesion and extravasation (25). This was associated with increased endothelium-platelet interactions and spontaneous thrombus formation in veins (26). Importantly, platelet depletion in ADAMTS13 deficient mice reduced leukocyte rolling in unstimulated veins suggesting that platelet and endothelial VWF promote leukocyte arrest thus facilitating thrombus formation (25).

### Activation

Sustained activation of endothelial cells by inflammatory stimuli, including circulating pathogen-associated molecular pattern (PAMPs), DAMPs, cytokines, chemokines, complement proteins and ROS cause alteration of the endothelial function (27, 28). The end result is an impairment of endothelial anti-coagulant, anti-inflammatory and immune-dependent properties, which are hallmarks of endothelial dysfunction (Figure 1). Experimental models of VTE showed that interferon-γ (IFN γ), transforming growth factor-β (TGF β), tumor necrosis factor-α (TNF α), interleukin-6 (IL-6), IL-17, IL-9, IL-1β contribute to thrombus formation and resolution through endothelial activation. Chemokines such as CXCL8/CXCL1 and CCL2 are also involved (29). It is important to note that endothelial cells interact with the complement system via the expression of specific receptors (C1q, C3a or C5a receptors) and complement proteins (C1, C3, C5, factor B) further contributing to inflammation (30). Lastly, oxidative stress mediated by ROS induce several signaling pathways leading to endothelial dysfunction (31).

Due to its localization, the endothelium plays a crucial role in supporting thrombus formation and resolution in collaboration with platelets and leukocytes. During the initiation phase of thrombus formation, endothelial cells, platelets and leukocytes (especially neutrophils and monocytes) activate each other leading to a highly pro-coagulant environment and the recruitment of additional blood cells. Interactions and mutual activation during thrombo-inflammation between endothelial cells, neutrophils, monocytes and platelets has been extensively reviewed elsewhere (32–35). Briefly, leukocyte recruitment to regions of thrombo-inflammation is primarily initiated by direct interaction between leukocytes and the inflamed endothelium. The activated endothelium expresses P-selectin allowing the rolling and the adhesion of neutrophil via the P-selectin glycoprotein ligand-1 (PSGL1). The upregulated expression of VCAM-1 (vascular cell adhesion protein) and ICAM-1 (intercellular adhesion molecule) allows a stronger adhesion of neutrophils on the endothelial surface. A key process linking inflammation to thrombosis is the formation of neutrophil extracellular traps (NETs) by diverse stimuli including cytokines, activated platelets via P-selectin, DAMPs and ROS. NET formation is conditioned to chromatin decondensation *via* H3 and H4 histone citrunillation by peptidylarginine deiminase 4 (PAD4). NETs trigger coagulation *via* the release of enzyme including neutrophil elastase (NE), cathepsin-G or myeloperoxidase (MPO). This induces FXII activation and inactivation and degradation of TFPI and TM. NETs also contain TF and favor platelet recruitment and activation via VWF, histones, fibrinogen and fibronectin (1, 36–38).

The inflamed endothelium releases WPBs containing VWF, P-selectin and other pro-coagulant and pro-inflammatory components (cytokines and chemokines) in the extra-cellular environment (23). Platelet adhesion and activation are related to integrin binding on endothelial cell surface. Interaction of GPIbα with VWF is crucial for platelet accumulation along the inflamed endothelium mostly in arterial thrombosis. Hence, high shear stress exposes A1 domain of VWF multimers, important for GPIbα binding. This mechanism was also found to drive the onset of thrombus initiation in venous thrombosis in the stenosis model of VTE. To explain these interactions, it was suggested that GPIbα interacts with VWF multimers with pre-exposed A1 domain (39). Platelet GPIIb/IIIa binds to fibrinogen, thereby crosslinking platelets with platelets and platelets with endothelial cells. Once activated, platelets release their granule content further fueling platelet and endothelial activation. Among these factors, selectins are important for platelet-endothelial and platelet-leukocyte interactions. In addition, platelet secrete several soluble factors affecting leukocyte function. These molecules including IL-8, platelet factor 4 (PF4), CCL5, CCL7, CCL3, CXCL4 and CXCL5 are important for the recruitment and tethering of neutrophils to the nascent thrombus in association with selectins. Importantly, enzymes secreted by the activated neutrophils mediate fibrin formation by platelets. The integrin CD11b/CD18, activated following neutrophil adhesion to platelet, further develops the pro-coagulant activity of activated platelets (9, 34, 40). Platelets also participate to neutrophil activation and NET formation. The reciprocal interactions between platelets and neutrophils have been demonstrated both in *in vitro* and *in vivo* experimental models including in VTE (2).

In parallel, monocytes are recruited to the inflamed endothelium through a CCL2/CCR2 mechanism and adhere to the endothelium through P- and E-selectin/PSGL-1 interactions. They initiate coagulation through TF expression, release of TF-positive microvesicles and interactions with platelets (41, 42). Monocytes/macrophages are also important for thrombus resolution by clearance of apoptotic and necrotic cells and matrix debris, profibrinolytic activity and thrombus neovascularization. Monocytes/macrophages are an heterogenous population of leukocytes. Importantly, it was recently suggested that monocyte conversion from pro-inflammatory Ly6Chi to patrolling Ly6Clo monocytes is important for thrombus resolution (43, 44). Finally, endothelial cell, platelet and leukocyte activation culminate to fibrin formation stabilizing the thrombus (Figure 1) (2, 45–48).

## Endothelial Sensing of Environmental Cues

Endothelial cells play critical roles in regulating immune functions including cytokine secretion, phagocytic function, antigen presentation, PAMPs and DAMPs sensing, immune cell trafficking and cell extravasation. Increased permeability of the endothelium during inflammation is an important underlying cause of almost all endothelial-related destructive sequelae to the vascular wall. Breakdown of the complex balance between pro- and anti-coagulant systems as a result of acquired disturbances often result in thrombosis.

**Figure 2:**
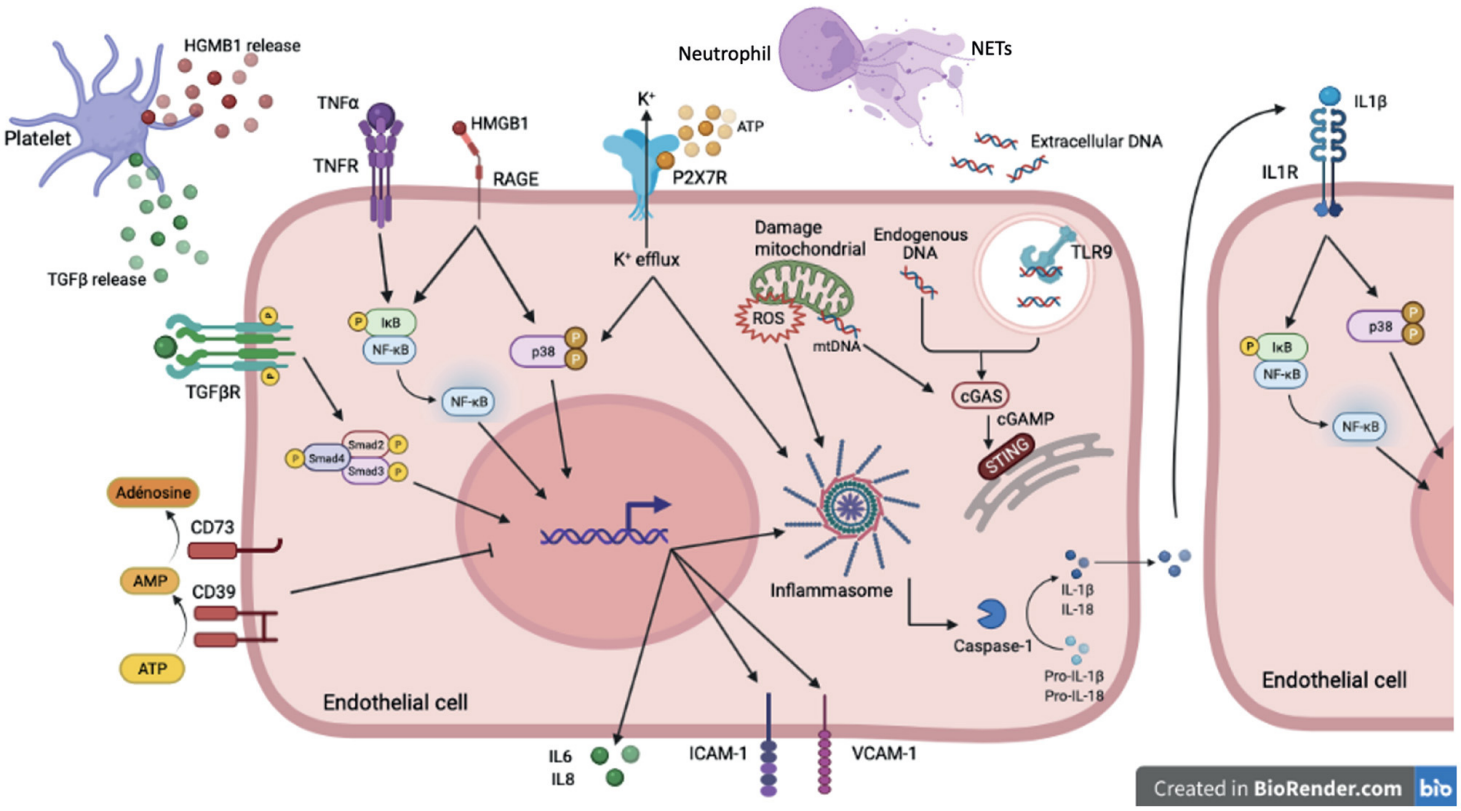
DAMP sensing by endothelial cells and signaling pathways induced downstream PRRs in sterile inflammation. In sterile inflammation, damage-associated molecular patterns (DAMPs) can activate pattern recognition receptors (PRRs). These extra- or intracellular DAMPs are recognized by many cell types including endothelial cells. Among PPRs, some are membrane receptor such as TLRs, cytokines receptors, purinergic receptor and some are intracellular receptor including cytoplasmic NLRP3 or reticuloplasmic STING. Cytokines (TGFβ, TNFα, IL-1β) activate via their receptors (TGFβR, TNFα R, IL1βR) many signaling pathways such as NFκB, p38 MAPK and SMAD. These pathways promote pro-inflammatory gene activation including IL-6 and IL-8 but also ICAM-1 and VCAM-1 involved in recruitment and adhesion of immune cells on the endothelium. The high-mobility group box 1 protein (HMGB1) mediates its pro-inflammatory effects through binding to RAGE and activation of MAP kinases and NFκB pathways. The assembly of the NLRP3 inflammasome, which allows the cleavage of the cytokines IL-1β and IL-18 into their active form, is activated by several stimuli. Binding of extracellular ATP to P2X7R results in Ca^2+^ influx and K^+^ efflux. K^+^ efflux can induce NLRP3 inflammasome activation. To counteract pro-inflammatory effects of ATP, the ecto-nucleotidase CD39/CD73 hydrolyzes ATP to adenosine thus preventing its binding to P2XR7. The reactive oxygen species (ROS) production by the mitochondria also contribute to NLRP3 inflammasome assembly. Extracellular DNA derived from cell damage can be recognized by TLR9. Intracellular and mitochondrial DNA activate cGAS signaling in the cytoplasm. Activation of cGAS induces the production of cyclic second messenger GMP—AMP (cGAMP), which binds and activates STING at the endoplasmic reticulum.

Danger molecules or alarmins such as DAMPs released during tissue damage in the extravascular environment are recognized by patterns recognition receptors (PRRs). Several type of PPRs can be activated including toll-like receptors (TLRs), NOD-like receptors (NLRs) but also non-PRR DAMP receptors including receptor for advanced glycation endproducts (RAGE) and ion channels (P2X receptors). Downstream signaling pathways activated by sterile inflammation lead to phenotypical changes in endothelial cells contributing to endothelial dysfunction (Figure 2; Table 1) (27, 28).

**Table 1:** Endothelial responses to molecules from the microenvironment and thrombo-inflammatory consequences.

**Molecular pattern**	**Receptor**	**Endothelial activation**	**Thrombo-inflammatory manifestations**	**References**
**Purine nucleotides**				
ATP	P2X7	↑ p38 phosphorylation ↑ IL-8, E-selectin	Production of cytokines and adhesion molecules Atherosclerosis	(40)
ATP	CD39	↑ NLRP3 ↑ IL-1β, TF ↑ Thrombus	Production of cytokines and activation of coagulation pathway Venous thrombosis	(39)
ATP	CD39	↑ IL-1β, IL-6, P-selectin ↑ Leukosequestration Neutrophil accumulation	Production of cytokines and adhesion molecules → leukocyte and neutrophil recruitment Venous thrombosis	(15)
**DAMPs**				
HMGB1	RAGE	↑ NFκB nuclear translocation ↑ phosphorylation ERK1 ↑ IL-8 ↑ ICAM-1, VCAM-1, E-selectin ↑ neutrophil adhesion	Production of cytokines and adhesion molecules → neutrophil adhesion on endothelium	(48)
HMGB1	RAGE	↑ phosphorylation ERK1, JNK, p38 Activation NFκB, Sp1 ↑ ICAM-1, VCAM-1 ↑ IL-8, TNFα, CCL2	Production cytokines, chemokines, and adhesion molecules	(44)
DNA mtDNA	STING	↑ Type I interferon pathway ↑ ICAM-1, VCAM-1 ↑ TF	Production of adhesion molecules and activation of coagulation pathway	(49–51)
RNA	TLRs	↑ TF ↑ permeability ↑ adhesion ↑ leukocyte recruitment ↑ angiogenesis	Activation of coagulation pathway, modification of endothelium metabolism → leukocyte recruitment	(52)
**Cytokines**				
IL-1β	IL1R	↑ oxidative stress Production of pro-coagulant mediator Production of IL-6	Production of cytokines, modification of endothelium metabolism Atherosclerosis	(53)
TNFα	TNFR	↑ P-selectin Production of ROS	Production of adhesion molecules, modification of endothelium metabolism	(54)
TNFα	TNFR	↑ TF, PAI-1 ↓Thrombomodulin	Activation of coagulation pathway, suppression of anti-coagulation effector → Acceleration of clotting time	(54)

*ICAM-1, inter cellular adhesion molecule-1; IL, interleukin; PAI-1, plasminogen activator inhibitor; RAGE, receptor for advanced glycation endproducts; ROS, reactive oxygen species; STING, stimulator of interferon genes; TF, tissue factor; TLR, toll like receptor; TNF, tumor necrosis factor; VCAM-1, vascular cell adhesion protein-1. ↑, Increase; ↓, decrease; →, leading to*.

### Purine Nucleotides and Inflammasome Activation

During tissue injury, purine nucleotides (ATP, ADP) are released in the extravascular environment. These molecules are recognized as danger signals by a variety of cells including endothelial cells, platelets, neutrophils and monocytes and activate inflammatory and thrombotic pathways (55, 56). The purine nucleotide metabolism is regulated by two systems that have opposite actions: CD73/CD39 that hydrolyzes ADP into adenosine has an anti-inflammatory and anti-thrombotic role; and P2X7 receptor, that binds ATP, activates the leucine-rich-containing family, pyrin domain containing 3 (NLRP3) inflammasome pathway and promotes pro-inflammatory and pro-thrombotic effects (15).

The inflammasome is extensively involved in non-sterile inflammation-dependent thrombosis. Recently, Wu et al. described a mechanism by which bacterial infection induces blood coagulation through activation of inflammatory responses by the host. They identified that inflammasome activation enhances the release of TF-positive microvesicles from macrophages in a mechanism dependent on pyroptosis. This ultimately leads to systemic activation of the coagulation (57). The importance of the inflammasome in different thrombotic-associated diseases along with infection or not has been reviewed elsewhere (53).

Activation of the inflammasome has also been demonstrated in sterile inflammation. Hence, microparticles from mice deficient for CD39 induce a pro-inflammatory and pro-thrombotic response from endothelial cells illustrated by increased expression of the adhesion molecules, ICAM-1 and VCAM-1, and the release of VWF and TNFα (54). More importantly, CD39 has an important protective role in venous thrombogenesis. Hence, haplo-insufficient mice develop much larger thrombus characterized by increased leukocyte recruitment, NET formation and increased fibrin and TF expression. Mechanistically, CD39 deficiency was associated with accumulation of extracellular ATP, activation of the NLRP3 inflammasome and subsequent IL-1β secretion (15, 55). Hence, when purine nucleotides are not metabolized by CD39, they might accumulate in the circulation and activate the P2X7 receptor expressed by endothelial or circulating cells leading to activation of NLRP3. Thus, CD39 has an important role in inhibiting NLRP3 and IL-1β signaling during VTE.

Additionally, activation of endothelial P2X7 induces p38 phosphorylation leading to IL-8, P-selectin and E-selectin expression in atherosclerotic plaques (56). The lack of P2X7 in mice abolished inflammasome activation and monocyte recruitment to atherosclerotic lesions (49). P2X7 receptors were also found to aggravate cardiac dysfunction in myocardial infarction, hypertension and diabetic retinopathy. Interestingly, P2X7 exerts pro-coagulant functions by inducing platelet activation and TF expression in macrophages, by enhancing the formation of the prothrombinase complex and by inducing the generation of phosphatidyl serine rich microvesicles. In an experimental model of arterial thrombosis, time to occlusion was delayed in P2X7 deficient mice compared to control animals (50). Since P2X7 is crucial for IL-1β production, this study suggests an important link between TF expression and activation of the inflammasome. Interestingly, it has recently been shown that in hypoxic conditions, such as exposure to high altitude, occurrence of thromboembolic events was increased. Thrombosis was the result of inflammation and enhanced expression of NLRP3, IL-1β, IL-18 and caspase-1 through a mechanism dependent of HIF1α (51). IL-1β release plays an important role in the production of additional pro-inflammatory mediators and upregulation of adhesion molecules on endothelial cells through autocrine and paracrine effects that would contribute to thrombus formation (53).

### High-Mobility Group Box 1

HMGB1 is a non-histone DNA-binding nuclear protein implicated in the regulation of DNA structure to facilitate transcription, replication, and repair (52). HMGB1, which is secreted by stimulated macrophages and activated platelets, is also one of the prototypical DAMPs. HMGB1 binds to RAGE, expressed on the endothelium, smooth muscle cells and monocytes/macrophages (52). Once release in the extracellular compartment, HMGB1 regulates leukocyte recruitment through RAGE-dependent production of cytokines, including TNFα, IL-6, CCL3, CCL4 and CXCL12, and expression of ICAM-1 and VCAM-1 (58). In a mouse model of VTE, HMGB1 accumulates at the endothelial surface as early as 1 h after induction of thrombosis. Increased HGMB1 deposition overtime was correlated with platelet-leukocyte aggregates. Pharmacological inhibition of HMGB1 resulted in significant decreased in thrombus size. Bone marrow or platelet transplantation experiments showed that platelets are the main source of HMBG1 thereby promoting monocyte recruitment and NET formation (59). In addition, Dyer et al. (60) confirmed that platelet specific deletion of HMGB1 reduced NET formation and corresponding thrombus size. Two studies further characterized the mechanism of action of HMGB1 by demonstrating that it is involved in increased cytokine secretion, especially IL-8. This was correlated with higher ICAM-1 and VCAM-1 expression, allowing neutrophil recruitment and adhesion to endothelial cells (52, 61).

### Reactive Oxygen Species

Oxidative stress is a major contributor to endothelial dysfunction in cardiovascular diseases. ROS have deleterious effects when produced in excess and participate in the production and secretion of cytokines linking them to inflammation and endothelial dysfunction. Major sources of ROS are NADPH oxidase, the mitochondria and eNOS (endothelial nitric oxide synthase) uncoupling. Hence, mitochondria are important contributors to intracellular ROS production and play a central role in endothelial function (62). As mentioned below, mitochondrial dysfunction and associated liberation of mitochondrial DNA (mtDNA) was shown to promote inflammation (63). In endothelial cells, mitochondrial ROS production is also amplified by activation of NLRP3 and in turns NLRP3 promotes ROS generation. NLRP3 activation increases the secretion of pro-inflammatory molecules, such as IL-1β, IL-18 and HMGB1, which might eventually lead to endothelial cell death through pyroptosis. In addition, eNOS uncoupling is an important characteristic of endothelial dysfunction. Hence, eNOS normally prevents platelet aggregation and adhesion. NO has also anti-inflammatory actions by inhibiting leukocyte interactions with the vessel wall, thereby reducing pathological inflammation and thrombosis. Importantly, in an experimental model of VTE, thrombus burden was reduced by inhibition of TF expression consequent to eNOS activation (64). Ischemic conditions created by the formation of thrombi in veins is also associated with the production of ROS from endothelial cells and recruited leukocytes. In turns, ROS promote the formation of NETs by neutrophils, induce expression of cytokines and adhesion molecules by the endothelium and TF expression by monocytes (9). Thus, ROS are involved in almost all deleterious effects promoting thrombosis and endothelial dysfunction including EndMT. ROS mediate their effect either by directly influencing TGFβ signaling or indirectly by activating NFκB and cytokine production. TGFβ is also inducing oxidative stress in endothelial cells through mitochondrial dysfunction (65).

### Cytokines and Chemokines

The role of cytokines and chemokines in VTE has been reviewed elsewhere (29). We will here only discuss IL-1β and TNFα that are important regulators of thrombo-inflammation and endothelial phenotype. IL-1β is involved in many pathologies related to inflammation and results from NLRP3 activation. IL-1β is produced by endothelial cells and platelets and directly affects endothelial functions. For example, IL-1β increases production of ROS, pro-coagulants mediators and impairs vasodilatation. IL-1β expression is initiated following activation of the endothelium by a priming signal such as TNFα leading to NFκB activation; and a second activation signal such as extracellular ATP or DAMPs. The first signal is important for expression of NLRP3, pro-caspase-1 and IL-1β and IL-18 in their inactive forms. The second signal allows the assembly of the inflammasome which leads to the cleavage of pro-caspase-1 into caspase-1. When pro-IL-1β is cleaved by caspase-1 to its active form, it is secreted in the extravascular environment and binds to its receptor. It has been shown in atherosclerosis that IL-1β directly activates endothelial cells and increases the production of thrombogenic mediators such as IL-6 (66).

TNFα is a central cytokine in the regulation of inflammation. TNFα has two receptors: TNF receptor type 1 (TNFR1) or TNF receptor type 2 (TNFR2). TNFR1 is ubiquitously expressed whereas TNFR2 is mostly expressed by immune and endothelial cells. TNFα receptor activation is associated with NFκB translocation to the nucleus and expression of pro-inflammatory genes (29). In presence of TNFα, the expression of P-selectin is upregulated on HUVEC surface allowing platelet rolling suggesting a pro-thrombotic role. Moreover, TNFα downregulates TM expression and increased TF and plasminogen activator inhibitor-1 (PAI-1) expression. All these endothelial changes might result in an acceleration of blood clotting induce by TNFα (67).

Importantly, IL-1β and TNFα are also potent inducers of EndMT. This will be discussed in the paragraph below together with TGFβ.

### Endogenous Nucleic Acids

As for purine nucleotides, intracellular DNA, RNA, rRNA and miRNA become disseminated into the circulation or retained at the site of cell activation and/or injury in the extracellular space. These extracellular nucleic acids elicit profound pro-inflammatory and pro-thrombotic effects. Analysis of extracellular DNA provided insight into their alarmin properties including highly pro-coagulant NETs and extracellular mitochondrial DNA, derived from cell injury and recognized as a DAMP by TLR9 to foster sterile inflammatory responses. Endogenous nuclear and mitochondrial DNA are also recognized by intracellular receptors including the stimulator of interferon genes (STING). STING is ubiquitously expressed in a variety of cells including endothelial cells. Recent studies demonstrated that STING is activated by mtDNA, free fatty acids and TNFα in endothelial cells resulting in control of immune cell transmigration (68–70). STING-induced endothelial dysfunction has been associated with elevated expression of endothelial inflammatory markers including TF and adhesion molecules. Importantly, thrombotic coagulopathy in COVID-19 patients have been attributed to STING over-activation and subsequent endothelial dysfunction in these patients (69). Despite a potential involvement in coagulopathy related to endothelial cell dysfunction, this mechanism has yet not been involved in VTE and would require future research.

As extensively studied by Klaus Preissner's group, different forms of extracellular RNA (eRNA), derived from activated or injured cells, are detectable in the extracellular compartment and contribute to the pathogenesis of cardiovascular diseases [reviewed in (71)]. Although, the exact nature of these eRNA remain to be defined, they contribute to a variety of mechanisms also involved in VTE. eRNA were first found to have pro-coagulant functions in arterial thrombosis by activating the contact phase pathway. It was later describe that they contribute to endothelial activation, leukocyte recruitment, vascular permeability, angiogenesis, macrophage polarization and cell death (72). A recent study showed that acute hypoxia induces TF expression in the vasculature in a TLR3-dependent manner. However, no functional characterization of the direct involvement of this pathway in VTE was investigated in this study (73). Table 1 summarizes endothelial responses to molecules from the microenvironment and which thrombo-inflammatory events are induced following endothelial activation.

## Endothelial Phenotypical Changes: Endothelial-to-Mesenchymal Transition

Endothelial dysfunction may be orchestrated by a process called EndMT. In this process, endothelial cells lose their endothelial markers to express instead mesenchymal markers. Endothelial cells also acquire mesenchymal characteristics including morphological changes, increased motility and cytoskeletal re-arrangements (74). They become more proliferative, thrombogenic and produce high amounts of extracellular matrix proteins such as fibronectin and collagen and express several adhesion proteins involved in leukocyte recruitment (75, 76). In addition, EndMT leads to altered endothelial cell junction organization, loss of cell polarity, increased cell proliferation and high invasive and proliferative potential (77). Specifically, when endothelial cells undergo EndMT, VE-cadherin, CD31, PECAM-1, EGF (TIE1 and TIE2), VWF and eNOS expression decreases and smooth muscle actin (SMA), smooth muscle protein 22-alpha (SM22α), vimentin, fibroblast specific protein-1 (FSP1), N-cadherin, type I collagen, fibronectin, nestin, CD73, matrix metalloproteinase- 2 and 9 (MMP-2 and MMP-9) and fibronectin expression increases (77–83).

Although, this is a physiological process during embryogenesis, in adulthood, EndMT is associated with various cardiovascular pathologies (78, 84). Hence, it was reported that EndMT is involved in the process of cardiac development and might be also important for the vascular system. However, when EndMT is induced in adult organisms, it is associated with atherosclerosis, pulmonary arterial hypertension, transplant arteriopathy, valvular disease and vein graft remodeling (84, 85). EndMT is induced by a number of factors and stimuli such as pro-inflammatory cytokines, hypoxia, abnormal mechanical forces but also as a secondary event following activation of pathways described in previous paragraphs by DAMPs. Multiple extracellular ligands are involved in the initiation and progression of EndMT programs (86). Also, epigenetic modifications might participate in EndMT and play an important role in cardiovascular diseases. DNA methylation, histone modifications and RNA interference are recognized as the most involved (82). However, EndMT is a complex process and due to the lack of standardization, relative expression of endothelial versus mesenchymal markers should be graduated and reversible or transient characteristics should be evaluated when one wants to show EndMT (87).

## Molecular Mechanisms and Regulation OF EndMT

### EndMT Inducers and Signaling Pathways

TGFβ is the most described inducer of EndMT but IL-1β, TNFα, IFNγ, hypoxia, high glucose and abnormal shear stress are also potent activators of this extreme phenomenon in the spectrum of endothelial activation (88). TGFβ binds with high affinity to type II serine/threonine kinase receptor (TGFβRII) and to activin-like kinases (ALK) 1 and ALK5 that are the predominant type I receptors (TGFβRI) in endothelial cells (76, 89). TGFβ signals through receptor complexes combining 2 type I and 2 type II components. Type I activation results in the activation of the canonical SMAD signaling pathway. Activated SMAD proteins form heterodimeric complexes with SMAD4 and translocate into the nucleus (82). There, they interact with various transcription factors such as Snail, Twist, Zeb and Slug and regulate the transcription of genes involved in EndMT (75). TGFβ might also induce non-canonical SMAD-independent signaling pathways including mitogen-activated protein kinase (MAPK; p38, JNK, ERK), phosphatidylinositol-3-kinase/Akt (PI3K/Akt), mammalian target of rapamycin (mTOR), Hippo/YAP, β-catenin/Wnt, protein kinase C and Rho-like GTPase (76, 78, 84). There are multiple signaling pathways induced by TGFβ alone or in combination with other inflammatory cytokines, including IL-1β and TNFα, hypoxia, abnormal shear stress or high glucose leading to EndMT (88, 90, 91). For example, during hypoxia the degradation of the hypoxia-inducible factor (HIF)1α is inhibited which then accumulates into the nucleus. Interestingly, HIF1α regulates the expression of Snail, Twist and ALK5 which might explain why hypoxic endothelial cells are sensitive to TGFβ signaling (76). Other cytokines can act as stimuli for EndMT, such as IFNγ, which acts via the Janus kinase (JAK) pathway and activation of STAT. Elevated HDL levels show anti-fibrotic effects by blocking the TGFβ/SMAD/Slug/ZEB signaling pathway. On the contrary, high glucose conditions have been shown to induce EndMT through the Akt/PI3K/NFκB pathway (82). Several studies showed that cytokine combination is more powerful in inducing EndMT. It has been particularly demonstrated for TGFβ, IL-1β and TNFα that converge to Sp1-dependent expression of EndMT-related genes (4). Since TGFβ, IL-1β, TNFα are released upon platelet activation along with other pro-inflammatory cytokines, it reinforces the important role of platelets in both thrombosis and inflammation and suggests that platelets participate in EndMT.

### Regulation of EndMT and Link With VTE

EndMT is a tightly regulated process in normal physiology. However, endogenous factors that inhibit EndMT have been less investigated. Several studies have highlighted the important role played by the fibroblast growth factor (FGF) in the regulation of TGFβ signaling (92). Hence, decreased FGF signaling leads to increased expression of TGFβ ligands and receptors and enhances TGFβ signaling resulting in EndMT (93). FGF controls EndMT and TGFβ signaling by maintaining high expression of Let-7 family of miRNAs, which downregulates ALK5 expression. However, in inflammatory conditions where FGF signaling is often reduced, the dramatic decrease of Let-7 miRNAs leads to rapid increase of ALK5 and amplification of TGFβ signaling. In addition, FGF directly inhibits ALK5-dependent expression of EndMT genes through activation of the Ras/MEK1 pathway (94). Accordingly, disruption of FGF signaling has been associated with aggravation of EndMT and atherosclerosis progression (95). BMP7 signaling is a second pathway that negatively regulates EndMT. It can antagonize TGFβ signaling through activation of SMAD1 leading to SMAD2/3 inhibition or by induction of ID proteins which can heterodimerize with SMAD2/3, thus inactivating their transcriptional activities (96).

Different types of non-coding RNA such as circular RNAs (circRNAs), long-non coding RNAs (lncRNAs) and miRNAs have been implicated in the regulation of EndMT. CircRNA are widely described as playing a role in epithelial-to-mesenchymal transition (EMT), a cellular transition process similar to EndMT and were found upregulated by 20-fold after TGFβ1 treatment (97). The circRNA DLGAP4 (DLG-associated protein 4) expressed in mice brain endothelial cells inhibits EndMT by interacting with miR-143 and thereby regulating expression of mesenchymal markers (98). Moreover, the CircHECTD1 was found to regulate the migratory capacity of endothelial cells by decreasing the expression of HECTD1 protein (99). The lncRNAs MALAT1 and GATA6-AS are also regulating EndMT. MALAT1 expression is increased in endothelial progenitor cells upon TGFβ1 treatment leading to downregulation of miR-145 and expression of SMAD3 and TGFβR2, thereby facilitating EndMT. GATA6-AS decreases TGFβ2-induced EndMT in human umbilical vein endothelial cells (HUVECs) by reducing SMA and calponin expression thus preventing VE-cadherin loss. GATA6-AS also interacts with LOXL2, which regulates endothelial gene expression via changes in histone methylation (H3K4me3) (97). However, little is currently known about how circRNAs and lncRNAs are regulated and functionally relevant in EndMT-related cardiovascular diseases.

Similarly, TGFβ signaling is directly controlled by miRNA that either downregulate growth factor receptor-bound 2 (Grb2), involved in fibrosis, (i.e. miR-200a) or the transcription factors Snail1 (i.e. miR-200b and miR-532) and Snail2 (i.e. miRNA-630) (84, 97, 100). TGFβ can also directly modulate expression of miRNAs that suppress inhibitors of mesenchymal gene expression or that affect endothelial gene expression. Normally, mesenchymal gene expression is kept inactive by transcriptional repressors SKI [SKI proto-oncogene (c-Ski)] and the ternary complex factor ELK1. SKI that inhibits the formation of the SMAD complex is the target of miR-155 and ELK1 is repressed by miRNA-27b thus inducing mesenchymal gene transcription. Finally, TGFβ increases the expression of miRNAs that suppress endothelial protein expression, including miR-21. PTEN is a target of miR-21 and an inhibitor of Akt which facilitates EndMT. Several other miRNAs were found to be modulated in EndMT but their precise role has not been defined yet (82, 100).

Interestingly, as for other cardiovascular diseases, miRNAs have been implicated in the pathogenesis of VTE. To date, only 4 studies have quantified miRNA in patients with venous thrombosis in an attempt to correlate miRNA expression to thrombotic events (101–104). Other studies have investigated miRNA expression profile in pulmonary embolism, recurrent venous thrombosis or experimental model of VTE. The most recent study in VTE patients showed that 9 miRNAs were significantly associated with venous thrombosis. Four of these miRNAs were found to regulate proteins of the coagulation cascade and might represent predictors of thrombotic events (104). Although this required further investigation, it has important clinical perspectives since miRNAs can be easily modulated. In addition, some miRNAs associated with VTE in these different studies were also found to be associated with EndMT in separate works (85, 105). For instance, miR-27b that promotes EndMT was found upregulated in pulmonary embolism. In chronic obstruction pulmonary disease, miR-145 was found to negatively regulate TGFβ signaling by decreasing SMAD3 phosphorylation, thus potentially interfering with EndMT (106). Interestingly, miR-145 was also associated with inhibition of TF expression thus preventing venous thrombosis in experimental models (107). These works suggest that miRNAs might link EndMT to VTE. Although, validation of causal implication of these miRNAs in EndMT-related VTE is required, it opens interesting research avenues.

## Epigenetic Regulation of EndMT

### Histone Modifications

Histone methylation, which is associated with both transcriptional activation and repression, is an important regulator of EndMT. EZH2 (enhancer of homologous zest 2) is the major histone methyltransferase responsible for the deposition of trimethylation marks on lysine 27 of H3 (histone 3; H3K27me3) leading to transcriptional repression. Co-stimulation of endothelial cells with TGFβ2 and IL-1β results in decreased expression of EZH2 and number of H3K27me3 marks at the SMA promoter. This induces SMA expression enabling EndMT. In addition, during cardiac development, histone deacetylase 3 (HDAC3) recruits EZH2 to prevent transcription of TGFβ1, and block physiological EndMT, which is an essential step to complete cardiac development. The association between HDAC3 and EZH2 might therefore be a mechanism involved in EndMT in cardiovascular diseases (97). Another histone demethylase that has been involved in the epigenetic control of EndMT is Jumonji domain-containing protein 2B (JMJD2B). This protein transcriptionally activates gene expression by demethylation of the repressive histone mark H3K9me3 and contributes to the methylation of the activating histone mark H3K4me3. JMJD2B is activated by EndMT-promoting stimuli including TGFβ1, TGFβ2, IL-1β and hypoxia. Using ChIP-sequencing, changes in H3K9me3 marks were found at various promoters upon induction of EndMT. Normally, expression of mesenchymal gene such as calponin is repressed by H3K9me3 marks. However, this repression can be reversed by JMJD2B-mediated demethylation following induction of EndMT (108).

Because aberrant HDAC expression and activity can promote EMT in cancer and that HDAC inhibitors prevent EMT, these proteins have been studied in the context of EndMT. Acetylation of H4 (histone 4) positively regulates SMAD3 upon combined TGFβ and Notch signaling stimulation. However, it remains to be defined which histone acetyltransferase (HAT) may be involved in this mechanism. Interestingly, treatment with TGFβ2 of cardiac endothelial cells induces histone acetyltransferase p300 expression, which is well known to be upregulated in fibrotic tissues. Therefore, p300 might be responsible for H4 acetylation and upregulation of specific SMAD3 target genes upon TGFβ1 stimulation (97). HDACs have also been studied in the regulation of gene expression during EndMT. As mentioned above, HDAC3 appears involved in repression of mesenchymal gene expression. In coronary endothelial cell lines, increased HDAC9 expression correlated with a generalized reduction of histone acetylation during EndMT. Importantly, class IIa HDAC inhibition prevented EndMT, whereas HDAC9 over-expression promoted EndMT. Endothelial-specific Hdac9 deficiency in mice was associated with reduced EndMT and a more stable atherosclerotic plaque phenotype (109). Others HDACs have been involved in EMT but their role in EndMT has not been studied yet.

### DNA Methylation

DNA methylation refers to the presence of methyl 1 groups on cytosine bases in the CpG islands of DNA. These CpG islands are mainly located in gene promoter regions, which are responsible for transcriptional activation. Addition of methyl groups via DNA methyltransferases (DNMTs) to these CpG islands is the most potent epigenetic regulatory mechanism to stably silence gene expression. Treatment with TGFβ1 of human coronary endothelial cells resulted in aberrant methylation of the promoter RASAL1 (RAS protein activator like 1) (a Ras signaling inhibitor), which contributes to EndMT (97).

**Figure 3:**
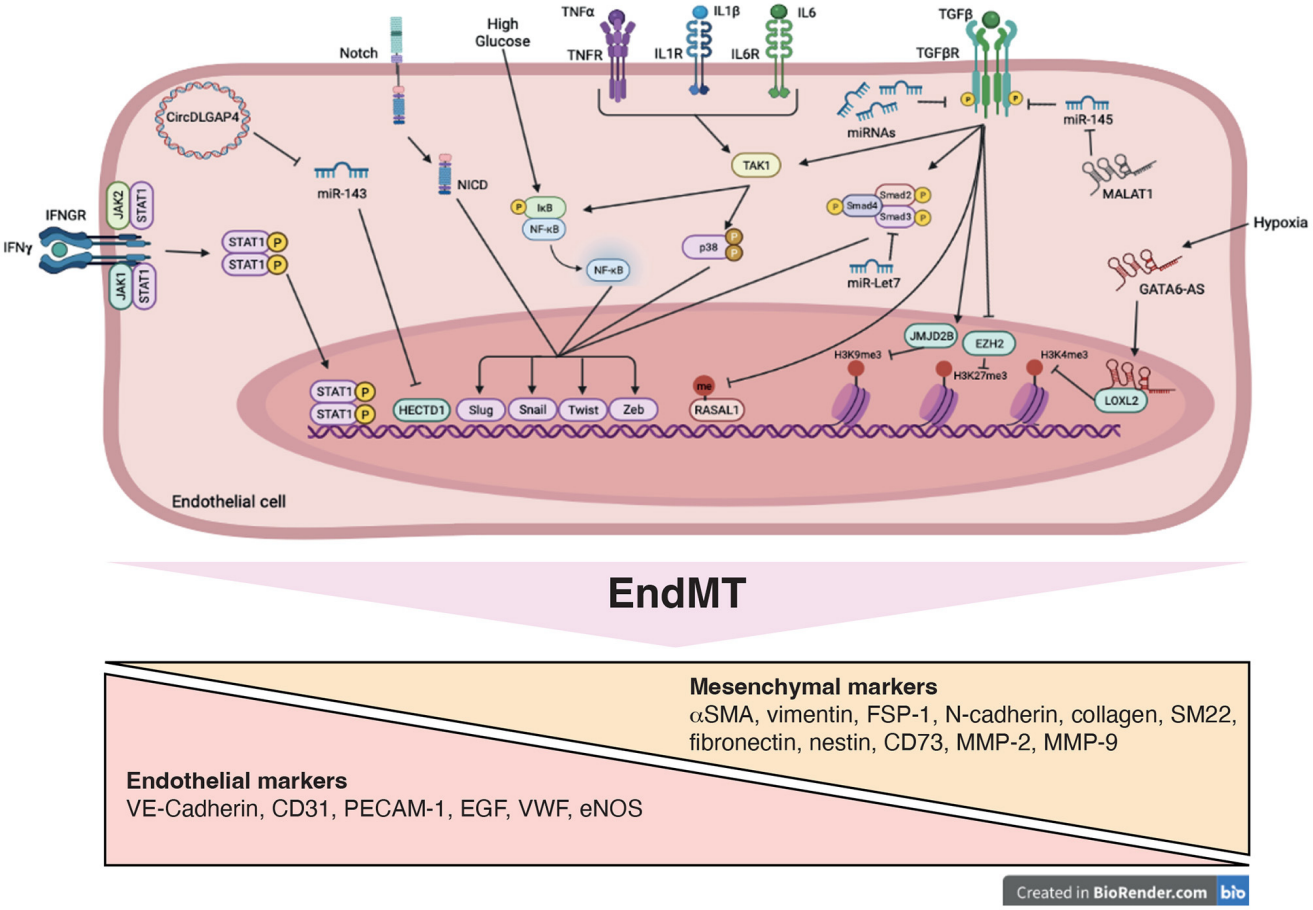
Signaling pathways and mechanistic drivers of endothelial-to-mesenchymal transition (EndMT). Stimulation with transforming growth factor-β (TGFβ), cytokines, Notch ligands, high glucose and hypoxia induce expression of transcription factors, such as Twist, Slug, Zeb and Snail resulting in EndMT. Epigenetic mechanisms including modifications of histone methylation marks and methylation of CpG island are induced. These processes are mediated by variety of actors such as microRNAs (miRNAs), long non-coding RNA (GATA6-AS and MALAT1), circularRNA (DLGAP4) and signaling pathways including SMAD, STAT, NFκB, and p38, promoting EndMT.

Regulation of EndMT involving epigenetic mechanisms to push toward expression of mesenchymal genes still requires investigation (Figure 3). A better understanding of the different regulatory networks involved might be useful to determine if maladaptive EndMT can be prevented or reversed to reduce or abrogate vascular remodeling. Aberrant regulation of epigenetic mechanisms is also strongly associated with inflammation and thrombosis. Some studies have investigated how DNA methylation and histone modifications were involved in VTE. Expression of several proteins from the coagulation cascade including FVII, FVIII and tPA was regulated by DNA methylation mechanisms leading to alteration of their plasmatic levels (110). Whole-blood DNA methylation analysis identified a potential association between methylation marks and quantitative biomarkers of thrombotic disorders (111). Regarding histone modifications, one particular histone modification, the citrullination, occurring in the formation of NETs have been extensively implicated in VTE. More importantly, studies have shown that the tPA gene expression was sensitive to histone modifications in endothelial cells and modified by HDAC inhibition (112, 113). *In vivo*, HDAC inhibition with valproic acid was associated with reduced thrombus burden supporting the idea that it can act as an anti-thrombotic agent (114). Elucidation of the epigenetic pathways involved in EndMT-related VTE will help in the discovery of new therapeutic targets.

## Other Factors Influencing EndMT

As discussed, TGFβ induces only partial EndMT and more potent effects are observed in association with other cytokines or stimuli including high glucose, oxidative stress, hypoxia or hypoxia caused by extreme environmental conditions. This implies that pathways independent of TGFβ might be activated during thrombo-inflammation that contribute to endothelial dysfunction and phenotypical changes. For example, thromboembolic complications were found to occur more frequently at high-altitude. The high altitude environment was associated with higher plasmatic levels of platelet and endothelial activation markers contributing to an hypercoagulative state (115). Hypoxia is also an important factor contributing to ischemic stroke and ischemia/reperfusion injury. In these pathological conditions, EndMT was found to contribute to the development of vascular fibrosis. In an experimental model of ischemic stroke, endothelial cells in infarct lesion undergo EndMT characterized by higher expression of TGFβR1, α-SMA, fibronectin, FSP-1, and Snail. Vascular brain damages due to EndMT were reversed by overexpression of the miRNA Let-7i (116). Additional miRNAs were found to regulate EndMT in the context of ischemic brain injuries, including the CircHECTD1 miRNA discussed above (117). In addition, it has been suggested that the endothelial dysfunction induced by the severe acute respiratory syndrome coronavirus 2 (SARS-CoV-2) might initiate pulmonary fibrosis and vascular remodeling through EndMT (118, 119). Although mechanisms by which EndMT is activated are still undefined, the interplay between hypercoagulopathy in COVID-19 patients and inflammasome activation might need to be further addressed (120).

## EndMT in Cardiovascular Diseases

Underlying pathological mechanisms in cardiovascular diseases involve multiple cell types, including endothelial cells, fibroblasts, smooth muscle cells, macrophages and other immune cells. Understanding not only how these various cell types interact during pathologic progression but also how the endothelial phenotype and more specifically EndMT are contributing is key to the molecular regulation of these diseases and has gain immense interest in the past years. The importance of EndMT in cardiovascular diseases has been recently reviewed elsewhere (96). Briefly, lineage tracing experiments have been used determine the contribution of EndMT to cardiovascular pathologies including atherosclerosis and pulmonary arterial hypertension (PAH) that will be discussed below.

### Atherosclerosis

Atherosclerosis is characterized by the accumulation of extracellular matrix proteins, mainly collagen and fibronectin, contributing to structural vascular remodeling, thickening of the arterial wall, and plaque formation. EndMT is thought to be one key process that leads to the differentiation of endothelial cells into pro-atherogenic cells leading to plaque formation (88). Recently, single-cell transcriptomic analyses supported evidence that EndMT features were observed in atherosclerotic plaque (121). Since, EndMT has been mostly associated and characterized in area of the vasculature exposed to disturbed flow where atherosclerotic plaque tends to form. Many sensors and pathways involved in transducing mechanical forces in endothelial cells contribute to endothelial dysfunction. Demos et al. recently identified Piezo, a mechanosensor that differentially modulates responses to laminar and disturbed flow. Piezo is a nonselective cation channel that activates PI3K-eNOS pathway which preserved the endothelial phenotype. On the contrary, disturbed flow activates the NFκB pathway contributing to the endothelial pro-atherogenic phenotype (122). The TGFβ-Alk5 is also important for shear-dependent activation of EndMT. Hence, endothelial-specific deletion of both TGFβR1 (Alk5) and TGFβR2 reduced plaque growth and induced plaque regression revealing an important link between EndMT and atherosclerosis (123). A direct connection between mechanical forces, mechanosensing and EndMT in the regulation of endothelial plasticity has recently been investigated. Results from this study showed that inhibition of Alk5 using siRNA in endothelial cells was associated with reduced flow-induced phosphorylation of SMAD2 and downstream expression of mesenchymal genes. Mechanistically, Alk5 association with Shc (Src homology and collagen) modulated EndMT and plaque formation in atheroprone areas (124). Other studies have confirmed that EndMT features were associated with plaque formation induced by disturbed flow, including expression and activation of Twist and Snail (125). It remains that other mechanosensors are probably involved in EndMT in atherosclerosis and would require further work.

### Pulmonary Arterial Hypertension

PAH is a complex pathophysiological adaptation characterized by vascular remodeling and neointimal thickening of pulmonary vessels with increased migratory, inflammatory and metabolically impaired states of smooth muscle cells following endothelial dysfunction and infiltration of immune cells. Direct link between PAH and EndMT has been evidenced in human and experimental models of pulmonary hypertension (126). Mechanistically, studies have demonstrated that EndMT is induced by several factors in the context of PAH including hypoxia, inflammation, TGFβ signaling and ROS production. As mentioned, hypoxia induces HIF1α that can directly bind to Twist and promotes the expression of mesenchymal genes. However, it was found that silencing HIF2α, which is highly expressed in endothelial cells from PAH patients, reduced Snail and Slug expression in association with increased expression of mesenchymal makers and decreased endothelial markers. Interestingly, endothelial-specific deletion of HIF2α prevented the development of hypoxia-dependent pulmonary hypertension in mice (127). However, it remains to determine if HIF2α deletion *in vivo* would rescue or prevent the development of EndMT. Pulmonary arterial endothelial cells appear to respond differently to IL-1β, TGFβ1, TGFβ2 than other vascular beds. Inflammation contributes to dysregulation of TGFβ-BMP signaling, which has been associated with predisposition to PAH. BMPR2 signaling in particular is responsible for the activation of SMAD1/5/8 that has antagonistic effects on TGFβ signaling. Loss of function mutations in BMPR2 gene are associated with increased EndMT-related expression of Twist1 and decreased expression of VE-cadherin. Reduced expression and function of BMPR2 is also associated with BMP9-induced EndMT through higher IL-6 expression. Interestingly, neutralizing IL-6 blocked BMP9 and consequent EndMT (96, 128).

## EndMT and VTE

Studies have also suggested that EndMT may be an important mechanism involved in long term complications of VTE. It is well established that thrombus resolution is associated with vascular fibrosis. However, mechanisms of vein wall fibrosis have only been partially characterized and the role of EndMT in these processes still require intense investigation. When studying the role of CCR7 signaling on vein wall fibrotic remodeling overtime in VTE models, Laser et al. found markers of EndMT in the vein wall. VTE was associated with increased expression of TGFβ, SMA, SM22, FSP-1, collagen I and III and MMP-2. Surprisingly, CCR7 from leukocytes confers protection against EndMT in VTE without affecting thrombus resolution. Although mechanisms of action of CCR7 were not elucidated, this study was the first to implicate EndMT as a potential explanation for fibrotic vein wall repair following VTE (129). Later, Bochenek et al. analyzed and characterized tissue sample from patients with chronic thromboembolic pulmonary hypertension (CTEPH) for structural and cellular composition. Along with the analysis of thrombosis resolution in mice, the authors proposed that fibrosis and scar formation in CETPH result from a sequence of events ranging from fresh to organized thrombus, myofibroblast and endothelial cell activation. These data suggest that fibrotic pulmonary lesions in CTEPH might result from unresolved thrombotic material. However, confirmation in experimental model using lineage tracing experiments would have definitely proved EndMT contribution to vascular fibrosis (130). Further mechanistic studies from this group demonstrated that endothelial TGFβ signaling and EndMT are important drivers of CTEPH. VTE was induced in mice with platelet-specific TGFβ1 deficiency and in mice with an inducible endothelial-specific deletion of TGFβ TGFβRII. The absence of TGFβ1 from platelets was associated with faster thrombus resolution. Surprisingly, endothelial-specific deletion of TGFβ RII significantly delayed thrombus resolution. Thrombi produced in mice with an endothelial-specific deletion of TGFβRII expressed characteristics of EndMT with increased fibrosis, collagen expression and CD31 positive cells co-expressing FSP-1 or SMA. In human CETPH specimen, immunohistochemistry analysis showed overactivation of TGFβ signaling characterized by higher Alk5/SMAD pathway activation. This was explained by higher circulating levels of TGFβ and overexpression of TGFβ. Interestingly, endothelin-1 was also overexpressed and blocking endothelin-1 receptor reversed EndMT and improved thrombus resolution. This study suggest that EndMT may be clinically relevant in CETPH patients contributing to obstruction of pulmonary artery branches with unresolved thrombo-fibrotic material (131). It was also suggested that EndMT might contribute to VTE. In a stenosis model of the iliac vein, thrombosis was associated with higher expression of mesenchymal markers and reduced expression of endothelial markers confirming previous observations. Interestingly, this study demonstrated that the vein wall of mice treated with rivaroxaban showed less markers of EndMT suggesting that anticoagulation therapy might reduce EndMT occurrence (132). These observations might have important repercussions when considering that fibrosis of the vein wall is potentially involved in enhanced risk of VTE recurrence. Additionally, when establishing a model of VTE recurrence in mice, Andraska et al. (133) observed that recurrent venous thrombosis was associated with important vascular remodeling with increased thickness of the vein wall and upregulation of TGFβ, IL-6, elastin and metalloproteinases expression. However, further investigations are still required to decipher precise mechanisms and chronological events occurring during VTE that promote the formation of vascular fibrosis following EndMT activation.

## Concluding Remarks

Reducing the deleterious impact of inflammation and endothelial dysfunction during VTE remains a major therapeutic challenge. This is not surprising considering that both mechanisms are intimately intricated and reflect complex interactions between immune and hemostatic responses. However, the possibility of reducing long term effect of thrombo-inflammation on the vascular wall would greatly prevent occurrence of PTS, CTED, CETPH and recurrent VTE. Endothelial dysfunction has been extensively studied in different cardiovascular diseases. The relatively recent discovery of the EndMT process highlighted the plasticity of the endothelial phenotype and have open new therapeutic perspectives. Understanding what is triggering EndMT might be as important as investigating mechanisms activated within the endothelium and how this translates into endothelial dysfunction and fibrosis. With accumulating evidence of the impact of EndMT in cardiovascular diseases, therapeutic strategies to prevent or reverse EndMT need to be considered.

A wide range of potential EndMT inducers are release during thrombo-inflammation. IL-1β, for example, represent an interesting target since it has been demonstrated in the CANTOS trial that its inhibition with canakinumab, might prevent recurrent cardiovascular events (134). Thus, future studies should consider similar strategies in the context of VTE. In addition, selective small P2X7 inhibitors were developed and may be used to block excessive IL-1β secretion during VTE as it has been suggested for atherosclerosis (49). IFNγ is also an inducer of EndMT. We and others have demonstrated that IFNγ was important for NK and T cell pro-coagulant activities in VTE. However, no studies have yet investigated if IFNγ was directly contributing to EndMT during VTE.

As recently reviewed by Choi et al., several drugs tested in clinical trials have been reported to inhibit EndMT in various animal disease models [see Table 3 of (65)]. These drugs block EndMT by targeting signaling molecules of the TGFβ pathway. An interesting strategy might be to prevent interactions between the endothelium, platelets and leukocytes that contribute to thrombosis, inflammation and EndMT. Severe coronavirus disease 2019 (COVID-19) infection is associated with endothelial pro-inflammatory and pro-thrombotic state that may be caused by release of VWF and P-selectin. Thus, a clinical trial is currently testing a monoclonal antibody targeting P-selectin, Crizanlizumab. This treatment might decrease thrombo-inflammation by blocking platelet and leukocyte interaction with endothelial cells thus reducing thrombo-inflammation (ClinicalTrials.gov Identifier: NCT04435184). Interestingly, given the interactions between coagulation and endothelial inflammation highlighted in this review, it is reasonable to assume that anti-coagulant therapies may have protective effects on endothelial phenotype. Heparin, a widely used anti-coagulant, have anti-inflammatory effects. Heparin and low molecular weight heparin (LMWH) can bind most of chemokines and cytokines. By competing with endothelial cell surface for cytokines, heparin and LMWH disrupts interactions between endothelial glycocalyx and cytokines thus preventing activation and trafficking of leukocytes (135). Antithrombin III might also exerts anti-inflammatory effects through upregulation of PGI_2_ by interacting with heparan sulfate on endothelial surface and inhibition of cytokines and TF production in endothelial cells and monocytes (136). New oral anticoagulants (NOACs), in particular rivaroxaban and dabigatran, show anti-inflammatory and endothelial protective effects. Rivaroxaban or dabigatran significantly reduced pro-inflammatory genes induced by thrombin in endothelial cells (137). Importantly, as mentioned above, rivaroxaban attenuates EndMT in a model of iliac stenosis (132). Alternative therapeutic strategies using the natural anti-coagulant properties of the endothelium have been developed. Thus, recombinant APC, TM and TFPI molecules were tested in experimental models and clinical trials (138). These molecules appear to modulate the endothelial inflammatory response that might potentially prevent interactions with platelets and leukocytes (Table 2).

**Table 2:** Major anticoagulant therapy efficacy, mode of action and potential effects on endothelial function.

**Anticoagulant**		**Action**	**Efficiency**	**Potential effects on endothelium**
Heparin	Unfractionated heparin (UFH)	Binding to antithrombin III (ATIII) → inhibition of thrombin, FXa and other clotting serine proteases.	Does not reduce mortality, organ damage, or hospital stay with increased risk of bleeding Beneficial effects on mortality in patients with sepsis-induced DIC	Anti-inflammatory effects: • Prevents binding of cytokines and chemokines to endothelial cells and consequent leukocyte recruitment. • Inhibits complement activation. (133)
	Low-molecular-weight heparin (LMWH)		Reduced sepsis severity, decreased 28-day mortality but increased bleeding	
ATIII	Kybernin P	Inhibition α-thrombin, FXa, FIXa, FVIIa, FXIa and FXIIa Binding endothelial GAGs → enhance PGI2 production	No significant reduction in mortality. Coadministration of heparin and ATIII exacerbates bleeding risk	Anti-inflammatory effects: • Increases PGI_2_: prevents platelet adhesion and activation. • Inhibition of cytokines and TF expression from endothelial cells and monocytes. (134)
NOACs	Rivaroxaban	Anti-FXa		Anti-inflammatory effects: • Reduces inflammatory genes induced by thrombin in endothelial cells (135). • Attenuation of EndMT in experimental model of VTE (130).
	Dabigatran	Anti-thrombin		Anti-inflammatory effects: • Reduces inflammatory genes induced by thrombin in endothelial cells (135).
APC	rhAPC: Xigris TM	Protein C binding α-thrombin → generate APC Soluble APC → Cleavage and inactivation FVa and FVIIIa	Preclinical model: reduction tissue damage and death Clinical trials: no reduction of death and increased risk for serious bleeding	Anti-inflammatory and cytoprotective effects: • Reduces thromboinflammation. • endothelial barrier stabilizing activities. • Cleavage of PAR-1 on endothelial cells induces protective genes.
	rAPC variants: 3K3A-APC	APC bound to endothelial protein C receptor → cleavage and activation of PAR1	Safer treatment	
Soluble rhTM	ART-123	Activation of PC Suppression of complement, endotoxin, and HMGB-1 protein	Less bleeding improved efficacy and safety in the treatment of DIC compared to heparin Phase 2b trials of sepsis patients: Lower D-dimer, prothrombin fragment and TAT concentration	Anti-inflammatory effects: • Complement and HMGB1 inhibition.
rTFPI	Tifacogin 1	Inhibition FXa and FVIIa/FT complex	Phase 2 trial: Reduction in TAT and IL6 level trend toward reduction in mortality phase 3 trial: not effect on mortality. Attenuated prothrombin fragment and TAT levels, leading to serious bleeding complications	Anti-inflammatory effects: • Reduces cytokine production.

*ATIII, antithrombin III; NOAC, novel oral anticoagulant; APC, activated protein C; TM, thrombomodulin; TFPI, tissue factor pathway inhibitor*.

It is unlikely that one drug may be used to treat all forms of EndMT in cardiovascular diseases. Because there is different kind of endothelium, it is highly probable that therapy would have to be adapted to specific features of each pathology. A better understanding of the mechanisms and regulatory networks controlling thrombo-inflammation-dependent development of VTE is still required.

## Author Contributions

MP, EO, VG-L, FC, and CL wrote the manuscript. All authors contributed to the article and approved the submitted version.

## Funding

This work was supported by Fonds de Recherche en Santé Respiratoire et Fondation du Souffle (Call 2019 to CL), Région Bretagne, Département du Finistère et Brest Métrople. CL also received funding from the Fédération Française de Cardiologie.

## Conflict of Interest

The authors declare that the research was conducted in the absence of any commercial or financial relationships that could be construed as a potential conflict of interest.

## Publisher's Note

All claims expressed in this article are solely those of the authors and do not necessarily represent those of their affiliated organizations, or those of the publisher, the editors and the reviewers. Any product that may be evaluated in this article, or claim that may be made by its manufacturer, is not guaranteed or endorsed by the publisher.
